# Mechanical properties of vertical-inclined pile foundation for onshore wind turbines

**DOI:** 10.1371/journal.pone.0323338

**Published:** 2025-06-04

**Authors:** Maogang Tian, Hechang Zhang, Shouhong Wang, Shilang Guo, Guangyuan Zhou, Shixin Ran, Bensheng Chen

**Affiliations:** School of Civil Engineering, Guizhou University, Guiyang, Guizhou, China; China Construction Fourth Engineering Division Corp. Ltd, CHINA

## Abstract

This study proposes an innovative Vertical-Inclined Pile Foundation to optimize the bearing performance of onshore wind turbine foundations. The Vertical-Inclined Pile Foundation (VIPF) and Vertical Pile Foundation (VPF) mechanical response mechanisms under wind turbine loading are compared based on the Hardening Soil (HS) model. Investigating the displacement distribution patterns, bearing platform deformations, pile internal force transfer, and pile-soil interaction laws of both foundation systems reveals the efficiency improvement mechanism of the VIPF. The results demonstrate that under identical loading conditions, the maximum displacement of the VIPF (20.63 mm) is 23.8% lower than that of the VPF (27.06 mm). The outer ring of inclined piles in the VIPF significantly enhances structural stiffness through spatial synergy, achieving uniform load distribution and effective redistribution of pile-body internal forces. Damage mode analysis indicates that the bearing platform primarily undergoes local tensile and compressive damage at the foundation ring-pile body connection, while the inclined piles establish an active pile-soil interaction system by strengthening soil confinement effects. Furthermore, parametric studies reveal that the pile base displacement exhibits a non-linear trend of initially decreasing and then increasing with larger inclination angles of the inclined piles. These findings provide a novel structural solution and theoretical basis for onshore wind turbine foundation engineering.

## 1. Introduction

With the transformation of the global energy structure, developing and utilizing new energy is increasingly becoming an essential direction for sustainable development. As a clean and efficient renewable energy source, wind power has been widely used onshore and offshore, and the wind turbine foundation, subjected to the coupled effects of vertical, horizontal, and moment loads [1], is a critical component of the wind turbine warp. In combating climate change and achieving sustainable development goals, realizing the innovation and application of the foundation of wind turbines under complex loading is an essential factor in improving wind energy in terms of effectiveness, economy, and utilization.

Many scholars have researched onshore and offshore wind turbines in recent years [2] proposed a simple and easily scalable loading device capable of applying multiple cycles and dynamic loading to a small model to evaluate the long-term performance of offshore wind turbine foundations [3] proposed future research directions for offshore coastal wind turbines and categorized and explained theoretical studies of structure-soil interaction [4] developed a three-dimensional finite element FE model to investigate the effect of long-term dynamic characteristics of monopile offshore wind turbine foundations under different loading cycles [5] investigated the dynamic characteristics of an offshore wind turbine with a three-legged suction hopper and found that strain measurements are an effective method to monitor the evolution of the dynamic characteristics [6] developed a three-dimensional numerical model and found that neglecting pile-soil interactions would result in fatigue damage to the OWT conduit frame of approximately 40%

[7] detailed the technology and cost trends for onshore wind energy and evaluated them concerning technical factors. [8] modified the insertion ring system of the foundation and, combined with field experiments, showed that the prestressing technique effectively limited the crack width and improved the section stiffness. [9] used nonlinear static simulations and verified that the model’s availability for soil and concrete foundations and the model parameters significantly affected the foundation performance response [10] proposed a new type of foundation with scaled-down modeling tests and presented several equations for calculating the foundation’s response under combined loads [11] established a simplified method for analyzing wind turbine towers’ resonance characteristics considering the pile foundation’s impedance. It was also found that the pile foundation significantly improved the resonance performance of the wind turbine structure [12] proposed a new cut-line pile barrel foundation for onshore wind turbines and carried out finite element tests and modeling tests. Compared with the traditional pile foundation, the SPBF can save about 40% of the footprint, about 22% of the steel reinforcement, and about 9% of the concrete [13]. Experimentally, the ring pile foundation was loaded in one direction, and the pile group’s bearing capacity and collapse mechanism under different load paths were obtained [14] proposed a new precast foundation for onshore wind farms. Compared with the enlarged foundation at the exact location, the new foundation uses 30.00% less concrete and 34.69% less steel.

In complex soil layers and mountainous areas, the wind turbine foundation usually adopts a pile foundation to ensure the foundation’s bearing performance in complex geological conditions. Contemporary wind turbine pile foundations still adopt the traditional circular straight pile group pile foundation, and many scholars have also proposed new wind turbine pile foundations [10,12], proving the new foundation’s economy and practicality. However, the form of pile foundations for onshore wind turbines is still scarce, and inclined piles are still not used for wind turbine foundations.

However, the traditional form of pile foundation remains as vertical piles. Compared with vertical piles, inclined piles have a more robust bearing performance. Moreover, many scholars [15–19] conducted field model tests and numerical model tests on inclined piles, analyzed the bearing performance of single pile and inclined piles under different conditions such as group pile action, uneven settlement action, seismic action, etc., and found that inclined piles have better-bearing characteristics. Moreover, to a certain extent, the bearing performance of inclined piles under different loads is related to the inclination angle.

**Fig 1:**
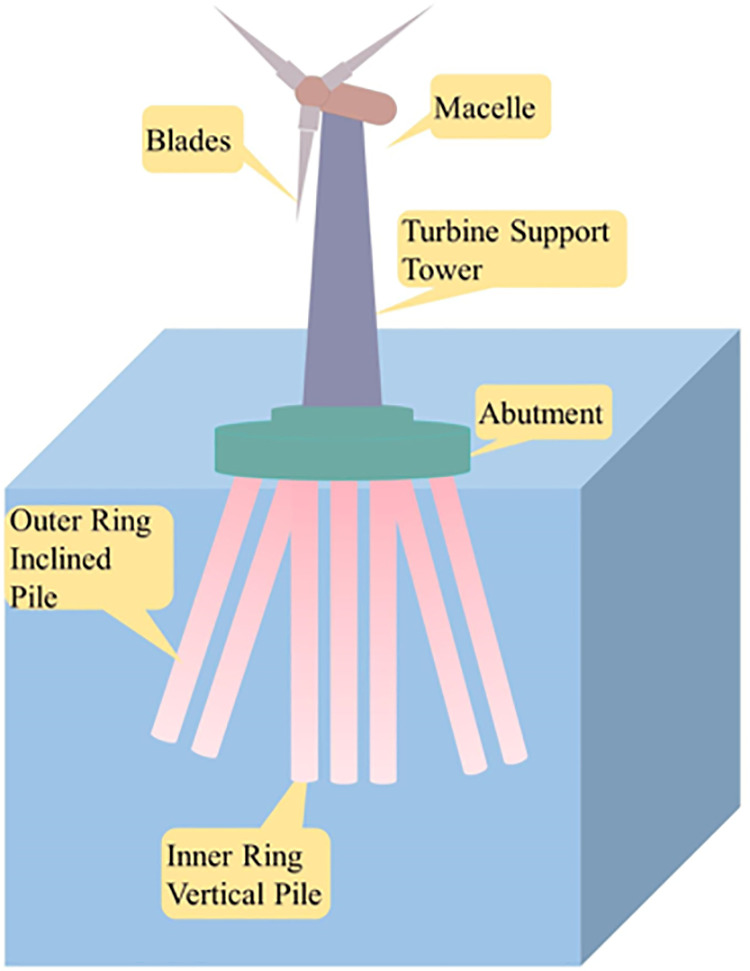
Schematic diagram of vertical-inclined pile foundation.

Given this, this paper proposes a new type of Vertical-Inclined Pile Foundation (VIPF) based on the high bearing capacity of slant piles. It combines vertical piles for the inner ring piles and inclined piles for the outer ring piles. As shown in Fig 1. The finite element numerical test is carried out to compare with the traditional Vertical Pile Foundation (VPF) and elaborate on the differences and advantages of the Vertical-Inclined Pile Foundation (VIPF) and the Vertical Pile Foundation (VPF).

## 2. Materials and methods

Plaxis3D is a geotechnical finite element analysis software for geotechnical works such as pits, tunnels, and slopes [20–22]. It has a variety of models for simulating soil changes, such as the commonly used Moore-Carron model (MC), soil hardening model (HS), and small strain soil hardening models (HSs). HS and HSs are more reflective of soil changes than MC, and the HSs model is more advantageous in describing soil deformation. Still, the soil parameters are more demanding and not easy to obtain. Compared with the HSs model, the HS model can also simulate soil deformation better, the parameters are easy to get, the calculation results will be more secure, and it has been used in various engineering practices [23,24].

The soil hardening model is a second-order high-level constitutive model that simulates the behavior of different types of soils (soft and hard) and is a hyperbolic elastoplastic model. The main parameters of the HS model are as follows: *E ref 50*: Standard triaxial drainage test cut line stiffness. *E ref oed*: Lateral Limit Compression Test Tangent Stiffness. *E ref ur*: Unloading/reloading stiffness. *m*: Stiffness-stress dependent power index, using a default value of 0.5. *c’*: Effective cohesion. *φ’*: Effective angle of internal friction. *ψ*: Shear angle*. R*_inter_: Interfacial friction coefficient.

### 2.1. Soil information

A typical soil layer was designed to carry out this test. There are four soil layers from the ground down: sandy soil 1, silty clay, sandy soil 2, and moderately weathered rock. HS is the intrinsic model for sandy soil 1, silty clay, and sandy soil 2. The medium-weathered rock is modeled as linear elastic with γ = 20.2 kN/m^3^, E = 600 MPa. In this study, the Transient Groundwater Flow (TGF) module in PLAXIS3D simulates transient groundwater behavior over time. The governing equations, derived from Darcy’s law and the principle of mass conservation, are solved numerically via the finite element method to obtain pore water pressure distributions. This approach explicitly accounts for the dependence of soil permeability on saturation variations. The groundwater table is set at -12 m below ground level. The drainage type is drained for sandy soils, medium-weathered rock, and undrained B for silty clay The soil parameters are as follows: Table 1.

**Table 1:** Soil modeling and mechanical parameters.

Soil layer	γ (kN/m^3^)	*E ref 50* (MPa)	*E ref oed* (MPa)	*E ref ur*(MPa)	*c’* (kPa)	*φ’* (°)	*v*	*R*_inter_	Depth (m)
sandy soil 1	17.8	4.5	4.5	13.5	0	26	0.20	0.8	-5
silty clay	19.2	8.0	6.5	19.5	22	24	0.20	0.7	-12
sandy soil 2	19.5	12.5	12.5	37.5	0	34	0.20	0.8	-25
medium-weathered rock	20.2	—	—	—	—	—	0.20	1.0	-40

### 2.2. Pile foundation

**Fig.2:**
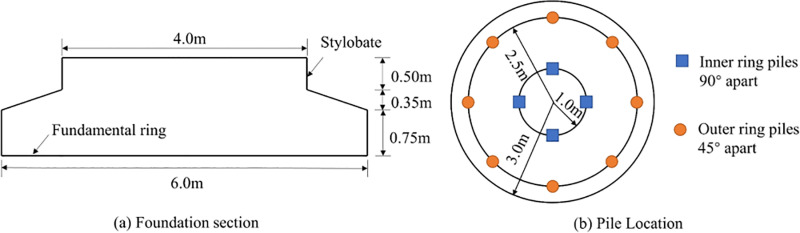
Pile foundation section.

Fig 2 shows a pile foundation section with a bearing platform consisting of a bottom foundation ring and an upper table column. The diameter of the foundation ring is 6m, the diameter of the table column is 2m, and the total height of the foundation is 1.6m, with circular piles arranged at radii of 1m and 2.5 m, respectively. The inner ring consists of 4 piles in a 90° circular arrangement, and the outer ring consists of 8 piles in a 45° circular arrangement.

**Fig 3:**
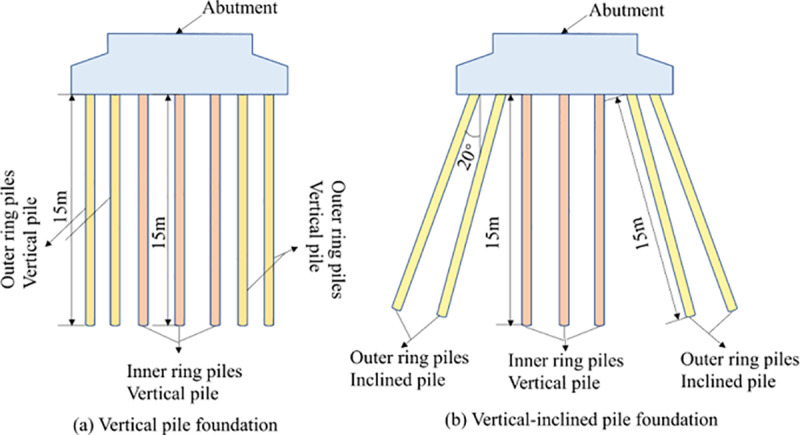
VPF and VIPF.

According to the arrangement of piles, they are VPF and VIPF. The inner and outer ring piles of the VPF are vertical piles, the inner ring piles of the VIPF are vertical piles, and the outer ring piles are Inclined piles, and the angle between the Inclined piles and the vertical axis is 20°, as shown in Fig 3.

The foundation ring and pedestal columns are made of C40 concrete. They are simulated using solid units weighing 25 kN/m^3^, a modulus of elasticity E of 32.5 GPa, and a Poisson’s ratio *v* of 0.15. The pile diameter is 0.4 m, the pile length is 15 m, and the material is C40 concrete, simulated by an Embedded Beam unit. The pile weight is set to be 6.0 kN/m^3,^ and the modulus of elasticity, E, is 32.5 GPa to avoid double calculating the weight between the pile and soil in Plaxis3D. The axial-lateral friction resistance of the pile is set to be related to the layer, the pile end reaction force is 200 kN, and the pile is set to be rigidly connected to the bearing platform.

### 2.3. Modeling and loading

**Fig 4:**
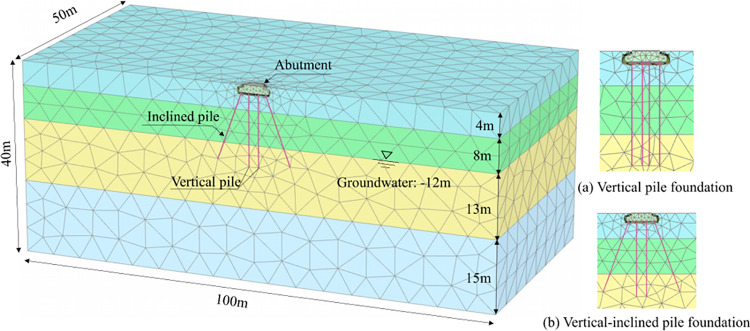
Model and meshes.

Based on the above information and the model’s symmetry, the half-plane strain models of VPF and VIPF are established, respectively. As Fig. 4. model size is 100 m × 50 m × 40 m with about 8300 cells and 13530 nodes. The model’s boundary conditions are set to be fixed at the bottom, regular to the front and back, and accessible at the top. In Plaxis3D, R_inter_ can be an excellent way to define the contact between the structure and the soil and set the contact roughness between the structure and the soil by changing the size of the R_inter_ in the material [25,26]. For this test, contact surfaces were created in the bearing platforms with a material pattern related to the adjacent soil, R_inter_, as shown in Table 1, to simulate the structure-soil contact action.

Plaxis3D provides direct loading of static and dynamic forces in each direction but does not provide direct loading of bending moments. In this test, the loading surface is set at the top of the table column, the rigid body unit is created, and the horizontal load, vertical load, and bending moment are applied by changing the parameters of the rigid body unit. The loading parameters are shown in Table 2.

**Table 2:** Loading mode.

Orientation	F_x_ (kN)	F_y_ (kN)	F_z_ (kN)	M_x_ (kN·m)	M_y_ (kN·m)	M_z_ (kN·m)
Loading	207	0	-315	0	5449	0

After the model was meshed, plasticity calculations were carried out for two different pile foundation conditions, with the following steps:

1. Generating the initial ground stress.
2. Activating the pile foundation and the contact surface.
3. Activating the load.

## 3. Analysis of results

### 3.1. Foundation displacement

**Fig 5:**
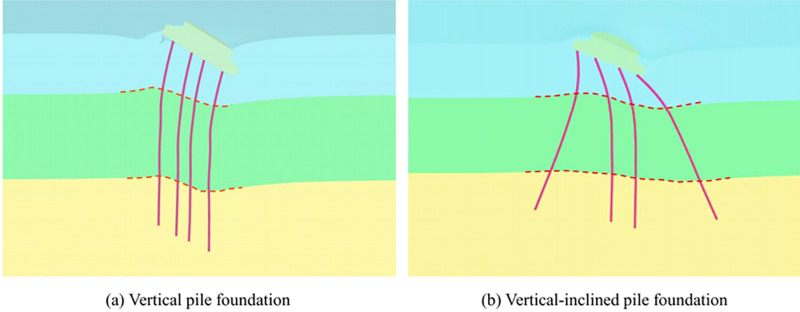
Morphing mesh.

There is a significant difference between displacement patterns in VPF and VIPF. Fig. 5 shows the deformed mesh for both model views magnified 100 times. Under the action of load, the VPF is tilted and damaged in the clockwise direction, and the pile body is bent and displaced in the cantilever type under the action of bearing platform pressing and soil resistance. The bearing platform drove the surrounding soil to sink and produced relative sliding, and the different soil layers in the buried piles all showed a sinking tendency.

Under the action of the same load, the bearing platform of the VIPF is tilted in the clockwise direction, which is consistent with the VPF, and its pile body shows cantilever bending displacement. In contrast, the deformation direction is opposite to that of the pile body of the VPF. There is settlement and heave in the deformation of the soil around the bearing platform, while there is heave in the deformation of the soil between the different soil layers where the piles are buried.

**Fig 6:**
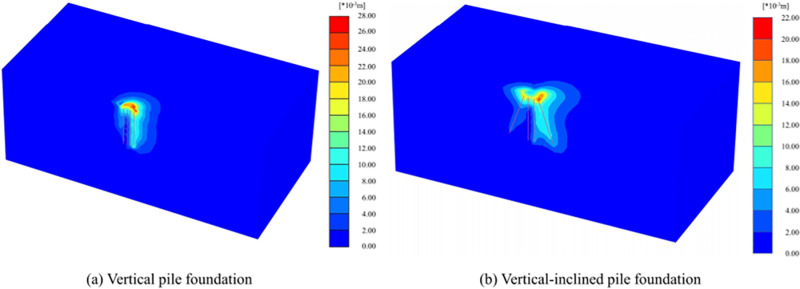
Displacement cloud maps.

Differences in displacement patterns make the maximum displacements different. As shown in Fig 6, the maximum displacements of VPF and VIPF are 27.06 mm and 20.63 mm, respectively, occurring at the bearing platform. The maximum displacement of the VPF is 1.31 times that of the VIPF, so the inclined arrangement of the outer ring of piles significantly reduces the pile foundation’s displacement. Compared with vertical piles, inclined piles are arranged with a certain degree of inclination, and the range of spatial effect between piles and soil is more comprehensive. Hence, the tensile bearing capacity is more robust [27].

The displacement of the VPF is concentrated in the bearing platform, which is lowered to a large extent, resulting in less deformation and influence of the surrounding soil and, thus, less interaction between the structure and the soil. The deformation of the soil body between piles is more regular, the changing trend is slight, the constraint of the soil body on the pile is small, and the influence factor is single. Hence, the bearing capacity of VPF mainly depends on the friction force between the pile and soil.

VIPF has a more vital interaction with the surrounding soil. Because of the inclined arrangement of the outer ring of piles, a larger conical space structure is formed between the piles and the soil, increasing the overall structure-soil stiffness. The inclined pile will be subject to the friction between the pile-soil in addition to the extrusion of the soil and gravity. The deformation of the soil between the piles shows a specific arch distribution under the interaction between the piles and the soil, which makes the constraint mode of the piles and the form of the force more complicated, thus reducing the displacement of the VIPF.

### 3.2. Deformation of bearing platform

**Fig 7:**
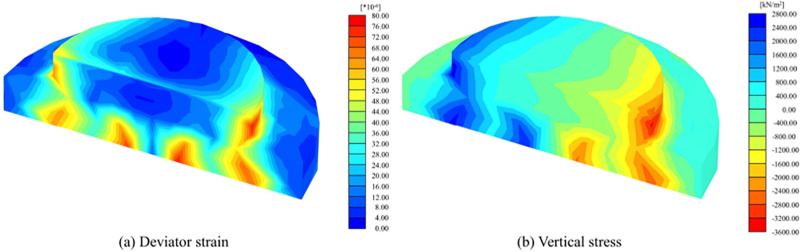
Strain-stress maps of vertical pile foundation.

Fig. 7 shows the deviatoric strain and vertical stress distribution of VPF. The deflection strains on the outside of the foundation ring and the top of the table column are minor, and more enormous deflection strains occur at the connection area between the foundation ring and the pile and on the outside of the table column. The vertical stress of the bearing platform contains tensile stress and compressive stress, which can be divided into tensile zone and compressive zone according to the distribution of stress, and the vertical stress is mainly concentrated in the connection between the foundation ring and the pile body and the outer side of the platform column. The stress in the foundation ring’s edge part and the platform column’s inner part is minor.

According to the distribution characteristics of partial strain and vertical stress, the damage mode of the VPF mainly exhibits integral-type tensile and compressive damage. The foundation ring and the pedestal column broke down under external loading. The damage was primarily concentrated at the pile connection area and the edge of the table columns, and these areas were subjected to high bias strain and stress concentration under loading, leading to the structural monolithic type of damage.

**Fig 8:**
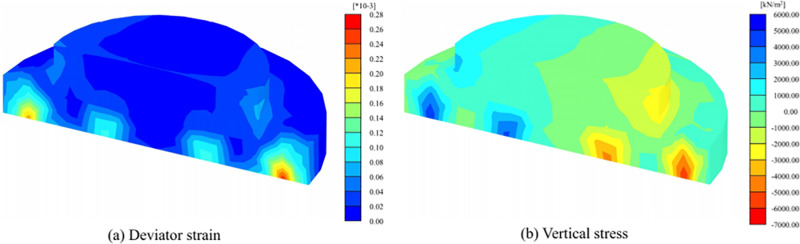
Strain-stress maps of vertical-inclined pile foundation.

Fig 8 shows the deviatoric strain and vertical stress distribution of VIPF. A considerable deflection strain was observed at the connection between the foundation ring and the inclined pile, compared to more minor deflection strains at other locations. It is clear that apart from the critical nodes, the deformation of the whole base structure is relatively homogeneous, with no apparent local strain concentrations. Under the action of external load, the vertical stress field of VIPF shows prominent tensile and compressive zones. The stress mainly concentrates on the connection between the foundation ring and the pile body. The deviatoric stress of the pedestal column in the VIPF is relatively tiny compared to the vertical stress, and there is no apparent destructive feature.

The damage mode of VIPF mainly exhibits localized type tensile and compressive damage. This damage mode refers to localized failure due to excessive tensile or compressive stresses in some critical regions. This localized damage pattern is relatively rare due to the structural design of VIPF with better distribution characteristics. Still, when it does occur, it is usually concentrated at the base ring-pile connection.

### 3.3. Pile internal forces

**Fig 9:**
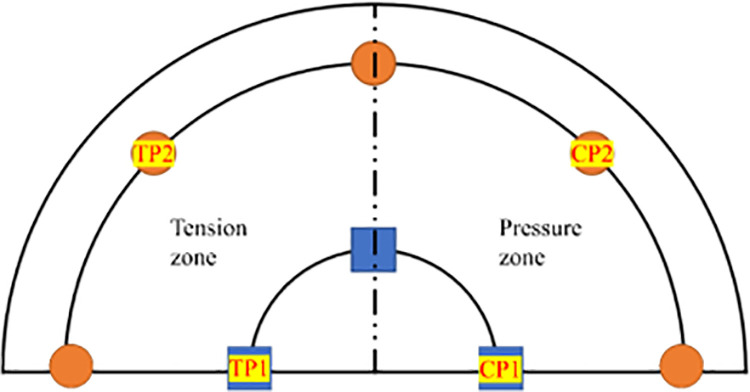
Location of monitored piles.

As shown in Fig 9, According to the force characteristics of the foundation, it is divided into a tension zone and a pressure zone. To better analyze the force characteristics of piles in different areas, two piles are selected in the tension zone and pressure zone parts in the inner and outer rings for internal force monitoring. The inner ring piles in the tension zone are TP1, and the outer ring piles are TP2. The inner ring piles in the compression zone are CP1, and the outer ring piles are CP2.

**Fig 10:**
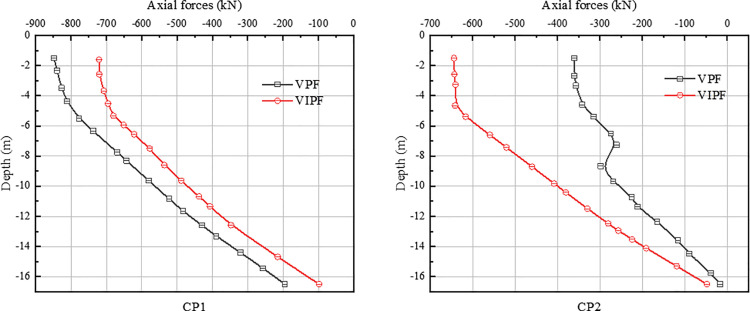
Axial force in the pressure zone.

**Fig. 11:**
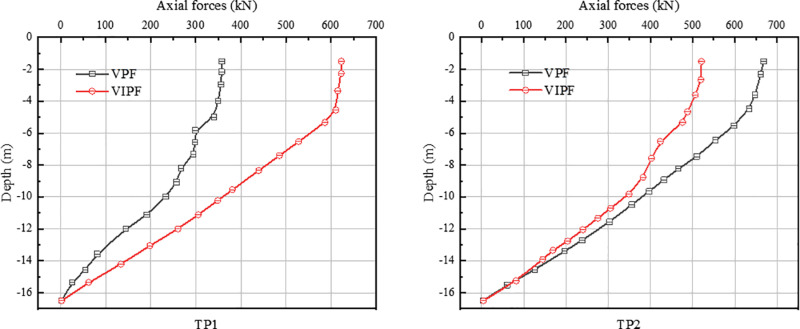
Axial forces in the tension zone.

Figs 10-11 shows the axial force distribution along the pile body for VPF and VIPF. The pile axial forces for both structures are maximum at the top of the pile and have a nonlinear trend of decreasing along the pile burial depth. The load will be applied directly to the pile-top location. As the pile penetrates deeper into the soil layer, the load will be gradually dispersed into the surrounding soil layer through the interaction between the pile and the soil layer.

In the compression zone, for the VPF, the maximum opposing axial force is 848 kN for the inner ring pile CP1 and 360 kN for the outer ring pile CP2, and the axial force of CP1 is 2.36 times that of CP2. Regarding pressure distribution, the VPF is mainly carried by the inner ring piles, while the outer ring piles carry less pressure.

For the VIPF, the maximum opposing axial force of inner ring pile CP1 is 720 kN, the maximum opposing axial force of outer ring pile CP2 is 664 kN, and the axial force of CP1 is 1.08 times that of CP2. The hostile axial forces of the inner and outer ring piles of the VIPF do not differ much, the distribution of the downward load is more uniform, and the axial forces of each pile body are similar.

In the tension zone, for the VPF, the maximum positive axial force is 358kN for the inner ring pile TP1 and 668kN for the outer ring pile TP2, and the positive axial force of TP2 is 1.87 times that of TP1. For the VIPF, the maximum positive axial force of the inner ring pile TP1 is 623 kN, the maximum positive axial force of the outer ring pile TP2 is 521kN, and the axial force of TP1 is 1.19 times that of TP2.

The difference between the axial forces of the inner and outer ring piles of the VPF is more significant, while the VIPF is more uniform in the distribution of the uplift load than the small VIPF. From the above two figures, it can be seen that for the VPF, there is a significant difference in the axial force distribution between the inner and outer ring piles. The maximum opposing axial force of piles in a VPF occurs in the inner ring piles, while the maximum positive axial force occurs in the outer ring piles. This unbalanced distribution of vertical loads may cause one part of the pile body to bear a more significant load. In contrast, the bearing performance of the other part of the pile body is under-exploited, resulting in insufficient overall bearing capacity of the foundation.

Unlike this, the VIPF changes the load distribution mode of the bearing platform by adopting the combination design of inner and outer ring pile bodies so that the axial force is no longer concentrated on a specific part of the pile body but bears the load uniformly as a whole, which realizes the uniform distribution of the axial force. The difference between the inner ring pile’s maximum positive and negative axial forces and the outer ring pile body is small. This balanced axial force distribution helps optimize vertical loads’ distribution, making the whole pile foundation system perform more reasonably and efficiently in bearing capacity and stability.

**Fig 12:**
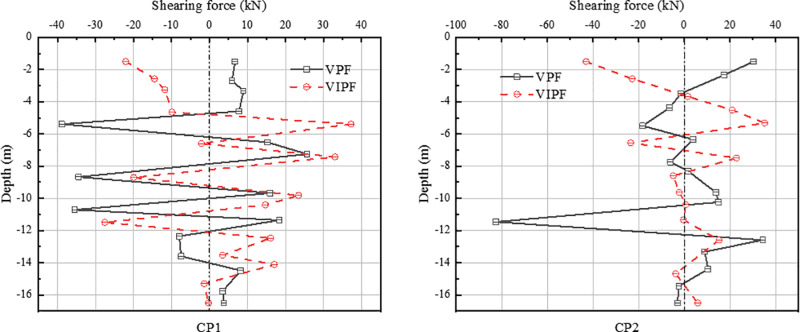
Shearing force in the pressure zone.

In the shear force diagram of the compression zone Fig 12, the shearing force of the inner ring CP1 pile exhibits a clear depth dependence. At a pile burial depth of 7 m or more, the shearing forces of VPF and VIPF are in opposite directions. From the top of the pile to the burial depth of 4 meters, the shearing direction of the VPF is the same as the horizontal displacement direction. In contrast, the shearing direction of the VIPF is opposite to the horizontal displacement direction.

The CP1 pile shearing for both pile foundations reached its maximum value at a burial depth of about 5m, and after that, the shearing trends converged. For the outer ring CP2 piles, the shearing force of the VPF varies considerably with the increase in pile burial depth, and its maximum shearing force occurs at a burial depth of 12 meters. In contrast, the maximum shearing of the straight-slope combination pile occurs at the top of the pile and decreases with increasing burial depth.

**Fig 13:**
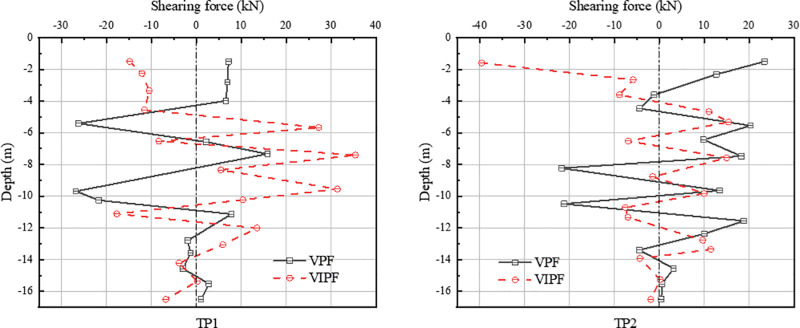
Shearing forces in the tension zone.

Fig 13 shows the shearing force diagram of the tension zone. The direction of TP1 pile shearing is opposite for both foundations, and the maximum shearing for the VPF is at burial depth -10m, while the maximum shearing for the VIPF is at burial depth -7 m.

For TP2 piles, the shearing force of the two types of pile foundations is in opposite directions from the top of the pile to a burial depth of -4 m. The distribution of the shearing force is gradually consistent with the burial depth. In contrast, the maximum shearing force of the VPF occurs at a burial depth of about 8 m, and the maximum shearing force of the VIPF occurs at the top of the pile.

There is a significant difference between the shearing forces of the two types of pile foundations, especially at the top of the pile, to the burial depth of 4 m. The pile shearing forces of the VPF and the VIPF show an opposite trend.

The shearing force of the VPF is in the same direction as the displacement of the pile foundation, indicating that it is mainly affected by external loads in this depth range. The opposite is true for VIPF, suggesting they are subjected to soil resistance more significantly than the external loads. This inconsistent shearing distribution characteristic reflects the fundamental difference in soil resistance between the two types of pile foundations.

This variability mainly stems from the inclined arrangement of the outer ring piles. It has been shown that the shearing capacity of inclined piles is generally higher than that of vertical piles under the same conditions [28]. Due to the inclined design of the inclined pile, a broader contact area is formed between the pile and the soil, which in turn enhances the interaction and spatial effect between the pile and the soil, resulting in a significant increase in the restraining effect of the soil on the pile.

Therefore, when the structure is subjected to external loads, the soil resistance of the VIPF will be higher than that of the VPF. The shearing force is redistributed due to the soil body’s restraining effect and the bearing platform’s joint action, thus forming a shearing force distribution law different from that of the traditional VPF.

Specifically, the VPF is subjected to less soil resistance in the upper part of the pile body, while the external load is relatively large. The VIPF effectively utilizes the inclined pile’s shearing characteristics and strengthens its upper part’s soil resistance, which makes the distribution of shearing force more complex and varied, forming a difference.

**Fig 14:**
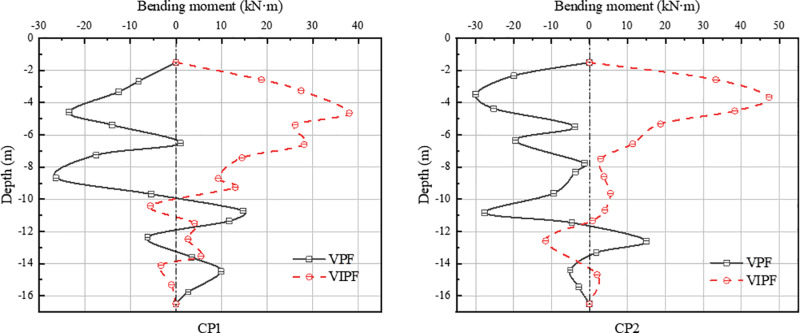
Bending moment in the pressure zone.

**Fig 15:**
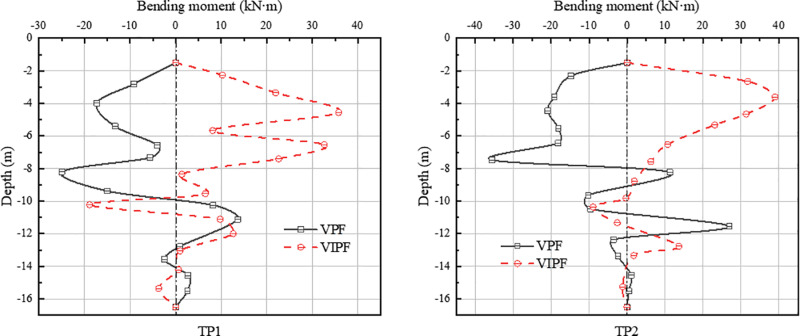
Bending moment in the tension zone.

Fig 14–15 shows the bending moments of inner and outer ring piles for both pile foundations. The distribution of bending moment along the pile body is more complex. Under the same working conditions, the monitored piles of both VPF and VIPF show opposite forms of bending moment distribution.

In the VPF, the maximum bending moment of the CP2 pile is at a burial depth of about 4m, and the maximum bending moment of CP1, TP1, and TP2 is at a burial depth of about 8m. The maximum bending moments of CP1, CP2, TP1 and TP2 are -26.37 kN·m, -30.01 kN·m, -24.95 kN·m and -35.60 kN·m, respectively. The maximum bending moment locations of the VIPF all occurred at a burial depth of about 4 m. The maximum bending moments of CP1, CP2, TP1 and TP2 were 38.02 kN·m, 47.31 kN·m, 35.77 kN·m and 38.99 kN·m, respectively.

From the bending moment distribution, it can be seen that the inclined arrangement of the outer ring piles not only changes the distribution pattern of the external load of the bearing platform but also changes the effect of the soil on the piles. The VIPF creates a different force mechanism due to the inclined setting of the piles, resulting in a change in the reaction force of the soil on the piles. The inclined arrangement of the outer ring piles allows the soil to act on the piles vertically and horizontally, increasing bending moments.

In the VPF, the shearing force is mainly concentrated in the middle and lower part of the pile body, resulting in a sizeable bending moment at a burial depth of about 8m. On the other hand, the VIPF has the maximum shearing force located at the top of the pile due to the inclination of the pile body, which can disperse the external load more effectively along the pile body and reduce the accumulation of bending moments in the middle and lower parts of the pile, so that the maximum bending moments appear at the burial depth of about 4m. This load distribution characteristic effectively reduces the force concentration of the pile foundation in the deep soil, thus improving the safety of the overall structure.

In addition, the sign of the bending moment (positive and negative values) reflects the operation of the two types of pile foundations under the same operating conditions. The negative bending moment of a VPF means that the pile tends to bend downward when subjected to external loads, which may trigger the uplift of the pile foundation. The VIPF, on the other hand, exhibits a positive bending moment, showing that the pile body is more adaptable to the load and can effectively resist the effect of uplift force.

### 3.4. Structure-soil action

Structure-soil interaction is an essential factor in the foundation’s ability to perform its load-bearing capacity, primarily in load transfer and in the extent of soil utilization around the pile.

(1) Load transfer and distribution

The VIPF optimizes the load transfer mechanism through a reasonable combination of straight and inclined piles. Compared with the single axial load transfer of the VPF, its load is more uniformly distributed between the foundation and the soil body, thus improving the overall performance of the foundation.

**Fig 16:**
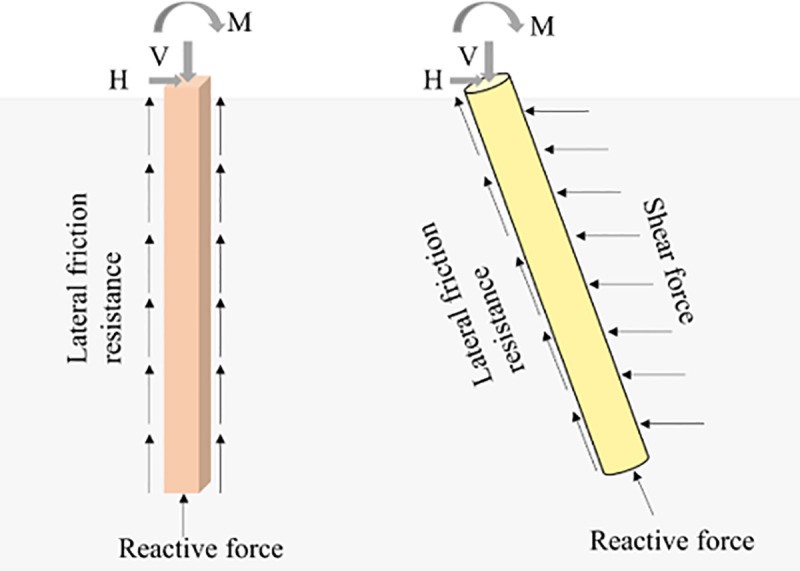
Sketch of forces on vertical and inclined piles.

As Fig 16, vertical piles are mainly subject to vertical loads due to their axial solid bearing capacity, and they can effectively transfer vertical loads to the deep soil. In addition to bearing axial loads, the inclined pile can bear greater horizontal loads and shearing forces. The inclined arrangement of the pile body enables it to effectively resist horizontal forces and lateral shearing forces, thus improving the overall stability of the foundation.

VIPF arranges a reasonable combination of vertical and inclined piles, giving full play to the advantages of these two kinds of pile bodies. Vertical piles carry mainly vertical loads, while inclined piles optimize the bearing and transfer of horizontal loads. This design approach improves the efficiency of load transfer and the distribution of loads between the foundation and the soil. The uniform load distribution effectively reduces the local stress concentration phenomenon in the soil body, avoids excessive compression or tension of the soil body, and improves the foundation’s overall stability and durability. Under the action of an inclined pile, it effectively resists lateral force and horizontal load, reduces the risk of overturning due to horizontal load, and enhances the foundation’s overturning resistance.

**Fig 17:**
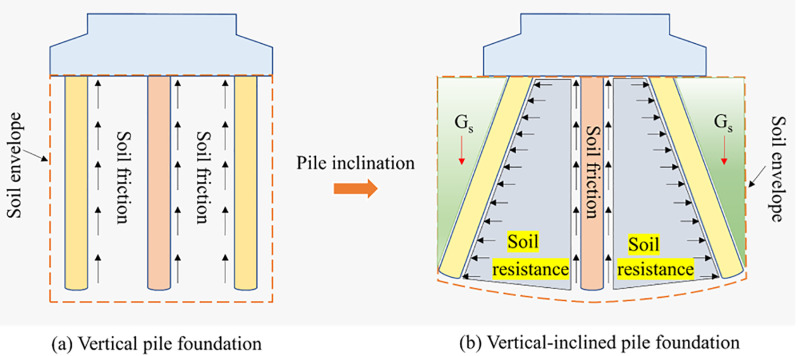
Pile-soil interaction.

In utilizing the soil around the pile, there is a significant difference between the VPF and the VIPF, which is mainly reflected in the characteristics of the soil influence envelope, as shown in Fig 17.

In VPF, the range of soil influence envelope is small, mainly relying on the friction between the pile and the soil to realize the load transfer and bearing. The bearing capacity of the foundation primarily depends on the friction characteristics of the pile and the surrounding soil.

In comparison, the utilization of peripile soil for the VIPF is significantly superior. The inclined pile’s introduction significantly enlarges the soil’s influence envelope. The inclined pile is subjected not only to friction between the pile and the soil but also to the influence of the soil gravity above the pile and the resistance of the soil between the piles. This design allows the VIPF to more fully utilize the bearing capacity of the surrounding soil and achieve a more efficient load transfer.

Therefore, a significant difference exists between VPF and VIPF regarding soil influence envelope and structural space action. When external loads are applied to a VPF, the soil around the pile depends mainly on the friction between the pile and the soil to transfer the load, and the nature of this friction is passive. The situation is even more complex for VIPF. The inclined arrangement of inclined piles changes the action of the soil body from a single passive response to an active one. A more uniform and effective load distribution is realized through the active soil support mechanism, which leads to an obvious advantage in the spatial structural effect. This active soil effect enables the VIPF to more fully utilize the soil around the pile to improve the stability and bearing capacity of the overall structure.

According to the force characteristics of the structure, the relationship between different inclination angles of inclined piles and the deformation of the structure under the same loading condition is investigated. The tests with inclination angles of 0°, 5°, 10°, 15°, 20°, 25°, and 30° are carried out respectively.

**Fig 18:**
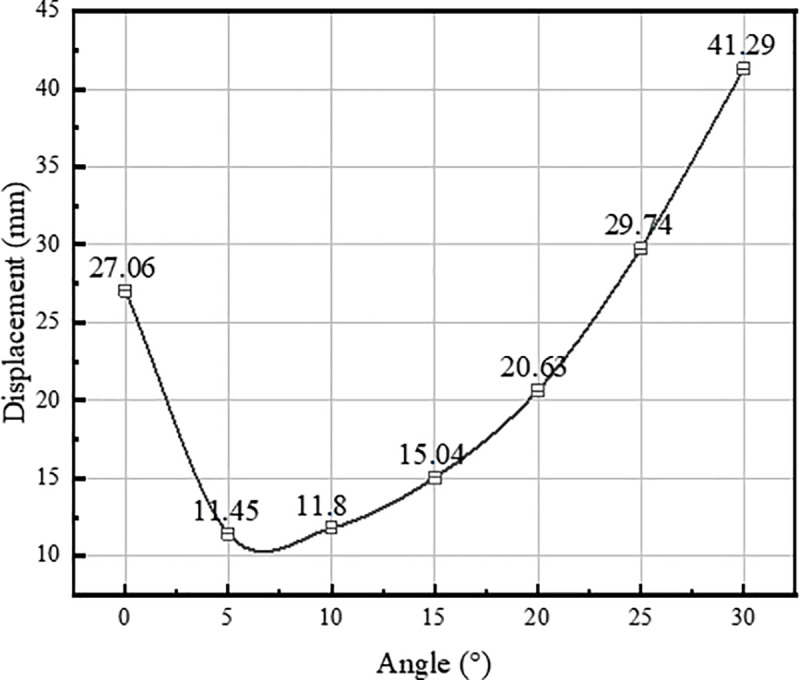
Displacement-inclination variation graph.

According to Fig 18, when the combination load is applied to the straight inclined combination pile foundation, there is a significant nonlinear relationship between the maximum displacement of the foundation and the inclination angle, and the displacement of the foundation tends to decrease and then increase with the increase of the inclination angle. The displacement of the foundation was 11.45mm when the inclination angle was 5° and increased to 41.29mm when the inclination angle was increased to 30°. This result shows that the inclination angle significantly affects the structural performance, and the appropriate inclination angle can effectively reduce the displacement of the structure. Excessive inclination may instead lead to unfavorable consequences and further increase the displacement of the structure.

This phenomenon is because the spacing between the inner and outer ring piles increases when the inclination angle increases, thus changing the soil resistance between the piles. Initially used as an integral member, the soil body between the piles becomes a single member at higher inclinations, reducing the foundation’s integrity and significantly reducing the foundation’s overall stiffness. In addition, the excessive spacing between the inner and outer ring piles also weakens the support effect and restraint of the soil between the piles, causing the foundation to exhibit a more excellent displacement response when subjected to complex loads and significantly higher moment loads.

## 4. Conclusion

In this paper, the bearing performance of different wind turbine foundations under complex geological conditions is investigated. By carrying out comparative tests of VPF and VIPF, the displacement pattern, bearing deformation, pile internal force, and pile-soil interaction mechanism of the two under wind turbine loading are analyzed in a systematic comparison, and the influence of inclined pile inclination angle on the mechanical performance of the VIPF is also explored. The main conclusions are as follows:

(1) The VIPF showed a clockwise tilting trend under the fan load, and the pile body underwent cantilever bending deformation, accompanied by differential settlement and bulging of the soil around the bearing platform. The maximum displacement of VPF is 27.06 mm, while the maximum displacement of VIPF is 20.63 mm, which is 23.8% lower. The inclined arrangement of outer ring piles can significantly reduce the displacement of pile foundations, and the VIPF has better anti-deformation performance than the VPF under the same working conditions.
(2) Under wind turbine load, compression and tension zones will be formed inside the pile foundation. According to the partial strain response and vertical stress distribution characteristics of the pile-soil system, the VPF bearing platform mainly exhibits overall tensile and compressive damage mode, and its damage area is centrally distributed in the connection area between the bearing platform and the pile foundation and the edge of the platform column; whereas the VIPF bearing platform exhibits localized tensile and compressive damage characteristics, and the damage core area mainly concentrates in the connection area between the foundation ring and the top of the pile.
(3) The VIPF redistributes the external load through the inclined arrangement of the outer ring piles, which is more uniform and reasonable than the VPF. The difference in internal force distribution between the two foundations is significant, with a large difference in the axial force of the pile body of the VPF and a similar distribution of the axial force of the pile body of the VIPF. The outer ring inclined pile changed the soil layer resistance above 4m, which led to the opposite direction of the foundation pile body shear force, the VPF shear force and displacement in the same direction, and the VIPF shear force and displacement in the opposite direction. Regarding the distribution of pile moment, the peak moment of the VPF is at the burial depth of 8m, while the VIPF is shifted upward to the burial depth of about 4m.
(4) The VIPF achieves optimum load distribution through the coordinated arrangement of vertical and inclined piles, with the vertical piles bearing the vertical loads and the inclined piles enhancing the bearing and transmission of the horizontal loads. The inclined piles’ design reconstructs the bearing platform’s load transfer path, which changes the soil action from passive to active and significantly improves the pile-soil interaction. Under the wind turbine load, the increasing inclination angle of the inclined pile will weaken the foundation’s overall rigidity and the soil’s supporting effect between the inner and outer rings, resulting in the nonlinear evolution of foundation displacement, which is firstly decreasing and then increasing.

## Supporting information

S1 File Minimal Data. (DOCX)
