# Supplementary material for: Mechanical properties of vertical-inclined pile foundation for onshore wind turbines
Source: PLoS One. 2025 Jun 4;20(6):e0323338. doi: 10.1371/journal.pone.0323338 (PMC12136306; doi:10.1371/journal.pone.0323338)
Supplement: S1 File — (DOCX) [file pone.0323338.s001.docx]

# Minimal data set

Table 1 Soil modeling and mechanical parameters

| Soil layer | γ (kN/m^3^) | *E ref 50* (MPa) | *E ref oed* (MPa) | *E ref ur*(MPa) | *c'* (kPa) | *φ'* (°) | *v* | *R*_inter_ | Depth (m) |
| --- | --- | --- | --- | --- | --- | --- | --- | --- | --- |
| sandy soil 1 | 17.8 | 4.5 | 4.5 | 13.5 | 0 | 26 | 0.20 | 0.8 | -5 |
| silty clay | 19.2 | 8.0 | 6.5 | 19.5 | 22 | 24 | 0.20 | 0.7 | -12 |
| sandy soil 2 | 19.5 | 12.5 | 12.5 | 37.5 | 0 | 34 | 0.20 | 0.8 | -25 |
| medium-weathered rock | 20.2 | **—** | **—** | **—** | **—** | **—** | 0.20 | 1.0 | -40 |

Table 2 Loading mode

| Orientation | F_x_ (kN) | F_y_ (kN) | F_z_ (kN) | M_x_ (kN·m) | M_y_ (kN·m) | M_z_ (kN·m) |
| --- | --- | --- | --- | --- | --- | --- |
| Loading | 207 | 0 | -315 | 0 | 5449 | 0 |

Table 10 Axial force in the pressure zone

| CP1 | | | |
| --- | --- | --- | --- |
| VPF | | VIPF | |
| Depth | Axial forces | Depth | Axial forces |
| -1.5 | -848.0957 | -1.59627 | -720.73096 |
| -2.3307 | -839.82538 | -2.57307 | -720.0686 |
| -3.48288 | -826.5954 | -3.6712 | -707.54169 |
| -4.37058 | -811.62518 | -4.5184 | -695.2038 |
| -5.49643 | -777.25601 | -5.32089 | -680.05822 |
| -6.3439 | -736.81262 | -5.93798 | -650.88696 |
| -7.75415 | -669.5 | -6.55508 | -621.48412 |
| -8.31183 | -642.88846 | -7.49886 | -577.45271 |
| -9.63315 | -579.85789 | -8.59719 | -535.8509 |
| -10.83927 | -522.25992 | -9.63088 | -488.11426 |
| -11.65925 | -483.03174 | -10.68377 | -437.95224 |
| -12.58777 | -429.62701 | -11.3364 | -406.85565 |
| -13.31611 | -388.91128 | -12.58532 | -347.34341 |
| -14.39453 | -320.98049 | -14.6832 | -215.20332 |
| -15.44727 | -256.52255 | -16.5 | -98.24902 |
| -16.5 | -194.99675 |  |  |

| CP2 | | | |
| --- | --- | --- | --- |
| VPF | | VIPF | |
| Depth | Axial forces | Depth | Axial forces |
| -1.5 | -360.61557 | -1.5 | -644.31653 |
| -2.67796 | -360.07666 | -2.57228 | -643.26453 |
| -3.33143 | -357.08691 | -3.25753 | -640.74034 |
| -4.59212 | -342.11572 | -4.64445 | -641.68329 |
| -5.38838 | -315.80246 | -5.3856 | -616.80341 |
| -6.51731 | -274.54492 | -6.59833 | -559.69794 |
| -7.25786 | -260.87009 | -7.42547 | -520.76592 |
| -8.6706 | -298.39966 | -8.7087 | -460.43227 |
| -9.6749 | -268.65488 | -9.8216 | -408.16589 |
| -10.70946 | -225.30378 | -10.40627 | -380.7269 |
| -11.35473 | -212.22527 | -11.49546 | -329.63025 |
| -12.35515 | -165.31767 | -12.47328 | -280.88431 |
| -13.5963 | -116.17227 | -12.94656 | -256.97105 |
| -14.48232 | -89.75506 | -13.53072 | -224.09306 |
| -15.75963 | -39.40978 | -14.11488 | -191.34508 |
| -16.5 | -16.93592 | -15.30744 | -119.31702 |
|  |  | -16.5 | -48.367 |

Fig. 10 Axial force in the pressure zone

Table 11 Axial forces in the tension zone

| TP1 | | | |
| --- | --- | --- | --- |
| VPF | | VIPF | |
| Depth | Axial forces | Depth | Axial forces |
| -1.5 | 358.33398 | -1.5 | 623.17834 |
| -2.15533 | 357.3692 | -2.26352 | 622.73267 |
| -2.95688 | 356.13304 | -3.3482 | 615.05927 |
| -3.97844 | 349.77618 | -4.55738 | 610.75818 |
| -5 | 340.35596 | -5.33252 | 586.52576 |
| -5.82106 | 298.99731 | -6.53372 | 528.04755 |
| -6.57449 | 298.42944 | -7.40241 | 485.73158 |
| -7.32793 | 295.02457 | -8.34415 | 439.76221 |
| -8.19986 | 267.19019 | -9.54686 | 381.242 |
| -9.07179 | 257.19421 | -10.21821 | 348.86521 |
| -9.96804 | 233.67032 | -11.1091 | 305.64093 |
| -11.12556 | 191.64285 | -12 | 260.57556 |
| -12 | 144.7606 | -13.05433 | 197.82178 |
| -13.57668 | 80.90746 | -14.21196 | 133.97393 |
| -14.56969 | 54.81817 | -15.35598 | 62.23155 |
| -15.34426 | 25.81169 | -16.5 | 2.12497 |
| -16.5 | 1.78866 |  |  |

| TP2 | | | |
| --- | --- | --- | --- |
| VPF | | VIPF | |
| Depth | Axial forces | Depth | Axial forces |
| -1.5 | 667.96851 | -1.5 | 521.09601 |
| -2.32059 | 661.29248 | -2.64898 | 519.8427 |
| -3.60575 | 647.8808 | -3.60249 | 506.55672 |
| -4.46568 | 633.87695 | -4.66438 | 487.90961 |
| -5.54194 | 598.16122 | -5.32089 | 476.1709 |
| -6.43241 | 554.4776 | -6.51795 | 423.77596 |
| -7.47196 | 509.6552 | -7.58038 | 402.46426 |
| -8.2283 | 466.41613 | -8.76642 | 382.64951 |
| -8.93244 | 431.92285 | -9.82156 | 349.50085 |
| -9.63659 | 397.43559 | -10.71244 | 305.76816 |
| -10.47806 | 356.20273 | -11.328 | 275.66225 |
| -11.55517 | 303.39111 | -12.04907 | 240.25064 |
| -12.71936 | 238.45622 | -12.77013 | 203.95862 |
| -13.38979 | 197.10518 | -13.34196 | 169.01546 |
| -14.55018 | 126.34447 | -13.91379 | 144.65238 |
| -15.52509 | 60.72085 | -15.25503 | 81.68448 |
| -16.5 | 3.20548 | -16.5 | 4.46409 |

Fig. 11 Axial forces in the tension zone

Table 12 Shearing force in the pressure zone

| CP1 | | | |
| --- | --- | --- | --- |
| VPF | | VIPF | |
| Depth | Shearing force | Depth | Shearing force |
| -1.5 | 6.7381 | -1.5 | -22.02282 |
| -2.67796 | 5.91896 | -2.57228 | -14.45502 |
| -3.33143 | 8.9264 | -3.25753 | -11.73805 |
| -4.59212 | 7.80048 | -4.64445 | -9.86087 |
| -5.38838 | -38.83108 | -5.3856 | 37.20984 |
| -6.51731 | 15.21088 | -6.59833 | -2.0949 |
| -7.25786 | 25.65656 | -7.42547 | 33.01365 |
| -8.6706 | -34.43893 | -8.7087 | -19.92969 |
| -9.6749 | 15.91363 | -9.8216 | 23.41053 |
| -10.70946 | -35.4992 | -10.40627 | 14.66452 |
| -11.35473 | 18.38506 | -11.49546 | -27.54309 |
| -12.35515 | -7.95196 | -12.47328 | 16.05127 |
| -13.5963 | -7.46591 | -13.53072 | 3.44015 |
| -14.48232 | 8.2403 | -14.11488 | 17.07407 |
| -15.75963 | 3.4559 | -15.30744 | -1.3924 |
| -16.5 | 3.78581 | -16.5 | -0.26825 |
|  |  |  |  |

| CP2 | | | |
| --- | --- | --- | --- |
| VPF | | VIPF | |
| Depth | Shearing force | Depth | Shearing force |
| -1.5 | 30.38594 | -1.5 | -43.10055 |
| -2.3307 | 17.58514 | -2.57307 | -22.81987 |
| -3.48288 | -1.49869 | -3.6712 | 1.56888 |
| -4.37058 | -6.4876 | -4.5184 | 20.99447 |
| -5.49643 | -18.2719 | -5.32089 | 35.25859 |
| -6.3439 | 3.92695 | -6.55508 | -23.5007 |
| -7.75415 | -6.06817 | -7.49886 | 22.85928 |
| -8.31183 | 1.71257 | -8.59719 | -4.8824 |
| -9.63315 | 13.82848 | -9.63088 | -2.18134 |
| -10.23621 | 15.03895 | -10.39103 | 0.67164 |
| -11.46111 | -82.68037 | -11.3364 | -0.38802 |
| -12.58777 | 34.47654 | -12.58532 | 15.2327 |
| -13.31611 | 8.97091 | -14.6832 | -3.7647 |
| -14.39453 | 10.40997 | -16.5 | 5.87163 |
| -15.44727 | -2.39041 |  |  |
| -16.5 | -2.98934 |  |  |

Fig. 12 Shearing force in the pressure zone

Table 13 Shearing forces in the tension zone

| TP1 | | | |
| --- | --- | --- | --- |
| VPF | | VIPF | |
| Depth | Shearing force | Depth | Shearing force |
| -1.5 | 7.16419 | -1.5 | -14.826 |
| -2.81067 | 6.93134 | -2.26352 | -12.06905 |
| -3.97844 | 6.5829 | -3.3482 | -10.4748 |
| -5.41053 | -26.224 | -4.55738 | -11.49263 |
| -6.57449 | 2.18729 | -5.66503 | 27.23976 |
| -7.32793 | 15.86751 | -6.53372 | -8.32909 |
| -9.68496 | -26.77419 | -7.40241 | 35.35795 |
| -10.25112 | -21.70019 | -8.34415 | 5.46566 |
| -11.12556 | 7.83582 | -9.54686 | 31.35465 |
| -12.78834 | -1.91513 | -10.21821 | 10.37457 |
| -13.57668 | -1.27363 | -11.1091 | -17.68201 |
| -14.56969 | -2.93697 | -12 | 13.50447 |
| -15.50865 | 2.80256 | -13.05433 | 5.93614 |
| -16.5 | 1.04638 | -14.21196 | -3.8687 |
|  |  | -15.35598 | 0.26667 |
|  |  | -16.5 | -6.77116 |

| TP2 | | | |
| --- | --- | --- | --- |
| VPF | | VIPF | |
| Depth | Shearing force | Depth | Shearing force |
| -1.5 | 23.36145 | -1.59627 | -39.52791 |
| -2.32059 | 12.6761 | -2.64898 | -5.78158 |
| -3.60575 | -1.13872 | -3.60249 | -8.85714 |
| -4.46568 | -4.38354 | -4.66438 | 11.03012 |
| -5.54194 | 20.29439 | -5.32089 | 15.3919 |
| -6.43241 | 9.84427 | -6.51795 | -6.88821 |
| -7.47196 | 18.24027 | -7.58038 | 14.96517 |
| -8.2283 | -21.72402 | -8.76642 | -1.47136 |
| -9.63659 | 13.35594 | -9.82156 | 9.96602 |
| -10.47806 | -21.19982 | -10.71244 | -7.58037 |
| -11.55517 | 18.81308 | -11.328 | -6.9724 |
| -12.35968 | 10.08039 | -12.77013 | 9.63659 |
| -13.38979 | -4.41752 | -13.34196 | 11.44224 |
| -14.55018 | 3.16595 | -13.91379 | -4.3101 |
| -15.52509 | 0.56468 | -15.25503 | 0.19481 |
| -16.5 | 0.44045 | -16.5 | -1.89101 |

Fig. 13 Shearing forces in the tension zone

Table 14 Bending moment in the pressure zone

| CP1 | | | |
| --- | --- | --- | --- |
| VPF | | VIPF | |
| Depth | Bending moment | Depth | Bending moment |
| -1.5 | -3.55454E-6 | -1.5 | 1.75629E-6 |
| -2.67796 | -8.19335 | -2.57228 | 18.78398 |
| -3.33143 | -12.59189 | -3.25753 | 27.47112 |
| -4.59212 | -23.51374 | -4.64445 | 38.02828 |
| -5.38838 | -13.98295 | -5.3856 | 26.1667 |
| -6.51731 | 0.94177 | -6.59833 | 28.12698 |
| -7.25786 | -17.50632 | -7.42547 | 14.48904 |
| -8.6706 | -26.36899 | -8.7087 | 9.26722 |
| -9.6749 | -5.46663 | -9.26515 | 12.99878 |
| -10.70946 | 14.73877 | -10.40627 | -5.62609 |
| -11.35473 | 11.64881 | -11.49546 | 4.07498 |
| -12.35515 | -6.24323 | -12.47328 | 2.63065 |
| -13.5963 | 3.48134 | -13.53072 | 5.47361 |
| -14.48232 | 9.89011 | -14.11488 | -3.32104 |
| -15.75963 | 2.68076 | -15.30744 | -0.99021 |
| -16.5 | 1.23086E-6 | -16.5 | -6.20581E-7 |

| CP2 | | | |
| --- | --- | --- | --- |
| VPF | | VIPF | |
| Depth | Bending moment | Depth | Bending moment |
| -1.5 | -6.14911E-6 | -1.5 | 4.87293E-6 |
| -2.3307 | -19.92484 | -2.57307 | 33.2866 |
| -3.48288 | -30.00274 | -3.6712 | 47.31438 |
| -4.37058 | -25.26669 | -4.5184 | 38.28958 |
| -5.49643 | -3.81091 | -5.32089 | 18.72463 |
| -6.3439 | -19.56667 | -6.55508 | 11.39539 |
| -7.75415 | -1.38272 | -7.49886 | 2.96568 |
| -8.31183 | -3.6987 | -8.59719 | 3.83479 |
| -9.63315 | -9.46533 | -9.63088 | 5.53878 |
| -10.83927 | -27.60419 | -10.68377 | 4.06548 |
| -11.46111 | -4.61679 | -11.3364 | 0.79545 |
| -12.58777 | 15.051 | -12.58532 | -11.55424 |
| -13.31611 | 1.81486 | -14.6832 | 2.01535 |
| -14.39453 | -5.03292 | -16.5 | -9.46609E-7 |
| -15.44727 | -2.83172 |  |  |
| -16.5 | -1.39146E-6 |  |  |

Fig. 14 Bending moment in the pressure zone

Table 15 Bending moment in the tension zone

| TP1 | | | |
| --- | --- | --- | --- |
| VPF | | VIPF | |
| Depth | Bending moment | Depth | Bending moment |
| -1.5 | -5.31527E-6 | -1.5 | 1.92754E-6 |
| -2.81067 | -9.16473 | -2.26352 | 10.26742 |
| -3.97844 | -17.29729 | -3.3482 | 21.94644 |
| -5.41053 | -13.29956 | -4.55738 | 35.76307 |
| -6.57449 | -3.99875 | -5.66503 | 8.11537 |
| -7.32793 | -5.64673 | -6.53372 | 32.62021 |
| -8.19986 | -24.95378 | -7.40241 | 22.58616 |
| -9.37837 | -15.04896 | -8.34415 | 1.35884 |
| -10.25112 | 8.23541 | -9.54686 | 6.52304 |
| -11.12556 | 13.58934 | -10.21821 | -18.86244 |
| -12.78834 | 0.92203 | -11.1091 | 9.75778 |
| -13.57668 | -2.44895 | -12 | 12.64325 |
| -14.56969 | 2.75903 | -13.05433 | 0.95991 |
| -15.50865 | 2.60576 | -14.21196 | 0.61015 |
| -16.5 | -7.46106E-7 | -15.35598 | -3.72063 |
|  |  | -16.5 | -1.7827E-6 |

| TP2 | | | |
| --- | --- | --- | --- |
| VPF | | VIPF | |
| Depth | Bending moment | Depth | Bending moment |
| -1.5 | 7.41617E-7 | -1.5 | 6.66592E-6 |
| -2.32059 | -14.78611 | -2.64898 | 31.7667 |
| -3.60575 | -19.14454 | -3.60249 | 38.99301 |
| -4.46568 | -20.89552 | -4.66438 | 31.39185 |
| -5.54194 | -18.07078 | -5.32089 | 23.09385 |
| -6.43241 | -18.10825 | -6.51795 | 10.68328 |
| -7.47196 | -35.60278 | -7.58038 | 6.25903 |
| -8.2283 | 11.3159 | -8.76642 | 1.94801 |
| -9.63659 | -10.2164 | -9.82156 | -0.26529 |
| -10.47806 | -9.61272 | -10.34804 | -8.95211 |
| -11.55517 | 26.92993 | -11.328 | -2.57206 |
| -12.35968 | -3.57669 | -12.77013 | 13.60863 |
| -13.38979 | -2.35558 | -13.34196 | 1.75836 |
| -14.55018 | 1.10103 | -15.25503 | -1.1375 |
| -15.52509 | 0.48995 | -16.5 | -7.30063E-8 |
| -16.5 | 1.87834E-6 |  |  |

Fig. 15 Bending moment in the tension zone

Table 18 Displacement-inclination variation graph

| Angle | 0 | 5 | 10 | 15 | 20 | 25 | 30 |
| --- | --- | --- | --- | --- | --- | --- | --- |
| Displacement | 27.06 | 11.45 | 11.8 | 15.04 | 20.63 | 29.74 | 41.29 |

Fig. 18 Displacement-inclination variation graph
